# No news may not be good news: A qualitative exploration of employee voice and leader responsiveness in Australian veterinary clinics

**DOI:** 10.1111/avj.70077

**Published:** 2026-03-26

**Authors:** HL Gray, E King, AM Neall, D Parker, RL Scotney

**Affiliations:** ^1^ School of Veterinary Science The University of Queensland Gatton Queensland Australia; ^2^ Student Services Directorate The University of Queensland St Lucia Queensland Australia; ^3^ Flinders Institute of Mental Health and Wellbeing Flinders University Bedford Park South Australia Australia

**Keywords:** disillusionment, employee voice, leadership, responsiveness, retention, veterinary

## Abstract

**Background:**

Retaining staff in veterinary clinics is an ongoing challenge, with workforce shortages threatening clinic functioning. The constructs of employee voice and leader responsiveness are reported as critical to employee retention, but their role in veterinary clinics is under‐researched. This study explores the influences, processes and outcomes of the employee voice and leader responsiveness interaction in Australian veterinary clinics to better understand how these interactions may support staff retention and inform practical strategies for clinics.

**Methods:**

Following purposive sampling, 24 semistructured interviews were conducted with current employees, leaders and owners of Australian general practice veterinary clinics to capture a range of relevant lived experiences. Interpretative Phenomenological Analysis was used to ensure a focus on foregrounding participants' voices during data exploration, providing sufficient depth within and breadth across groups.

**Results:**

Leaders' approaches to employee voice evolved over time, shaped by values and skills. Heavy workloads and constant pressure limited their capacity to respond effectively. Employees weighed perceived safety, ease and effectiveness before speaking up. Silence fed discontentment and low morale. When employees voiced, leader responses had a profound impact on how employees felt: ‘my back is covered’ and ‘grateful’ or ‘disrespected’, ‘dismissed’, ‘bitter’ and ‘unappreciated’.

**Conclusion:**

Early proactive voice met with constructive leader responses can address concerns and promote loyalty and enthusiasm, highlighting its potential to encourage ‘enthusiastic staying’ in veterinary clinics. Organisations should ensure employees can safely and easily speak up and equip leaders with the skills and capacity to effectively respond. Further research should examine contextual conditions enabling these interactions.

**Abbreviations**
AVA: australian veterinary association
EV: experienced veterinarian
IPA: interpretative phenomenological analysis
L: clinic leader
PET: personal experiential theme
RV: recent graduate veterinarian (up to 5 years' experience)
VN: veterinary nurse or technician

In 2018, industry data from the Australian Veterinary Association's workforce survey identified that veterinarians were leaving the profession at a rate that resulted in ‘critical gaps in the veterinary workforce’.^1^ More recently, the Australian Veterinary Association's 2023 workforce survey reported that veterinary clinics are still struggling to recruit staff, with 37% of clinics advertising for over 12 months, an increase from 24% in 2018.^2^ These figures are aligned with a 2023 industry white paper examining veterinary recruitment and retention, which highlighted urgency in tackling veterinary workforce issues.^3^

From an employee's perspective, consciously choosing to stay in a workplace is distinct from ‘not leaving’^4^ and there is increasing scholarly focus on cultivating the ‘right’ kind of staying where employees are ‘enthusiastic stayers’.^5^ There is a pressing need for greater understanding of what underpins or undermines ‘enthusiastic staying’ in the veterinary profession. In response, researchers are increasingly recognising how contextual and systemic factors such as leadership,^6^, ^7^ employer support,^8^ incivility^9^ and workplace conditions^10^ affect employee outcomes.^11^ A key factor in workplace dynamics is the interaction between leaders and employees, studied through the concepts of employee voice and leader responsiveness.^12^, ^13^, ^14^, ^15^

Employee voice can be defined as ‘informal and discretionary upward communication by an employee of concerns or suggestions to persons in a position to take action, with the intent to bring about improvement or change’.^16^ Following from this, leader responsiveness refers to how well leaders respond to employees' needs and concerns in an efficient and effective way,^13^ including leaders' willingness to reward and implement ideas once they are offered.^17^

Research in aligned sectors (i.e., helping professions) has shown that improved management of the interaction is linked with positive workplace outcomes including reduced employee intention to quit in the Australian service sector,^18^ reduced staff turnover in UK nurses,^15^ reduced burnout in Australian nurses,^13^ and increased engagement (dedication, energy and absorption in work) in UK private, public and not‐for‐profit employees.^14^ These positive outcomes are attributed to early intervention and positive management of employee concerns, before they escalate into major issues that cause disillusionment or discontentment. The 2021 AVA workforce survey identified that one third of veterinarians who were thinking of leaving the profession cited ‘disillusionment with the profession’ as the main reason.^19^ Therefore, improved communication that resolves concerns early in the process may be a valuable retention strategy. Although this association might seem self‐evident, the single study investigating employee voice in the veterinary sector found that British veterinary nurses would change clinics rather than speak up about concerns in their current clinic.^20^

Whilst extensive research about employee voice exists in other sectors, there is currently no literature on employee voice in Australian veterinary clinics. Hence, little is known about if and how employees speak up, how leaders respond, and if similar outcomes exist in this context. To address this gap, qualitative methodology was used to explore the lived experiences of employees, leaders and owners of Australian veterinary clinics, including the influences, processes and outcomes of the employee voice and leader responsiveness interaction. Qualitative research is increasingly valued in the veterinary domain as it offers the opportunity to understand complex phenomena and understand why people think, feel and behave the way they do in particular contexts.^21^

There were two research questions for this study. Firstly, how do employees in veterinary clinics make sense of their experiences around speaking up at work, and the response they receive from leaders? Secondly, how do owners or leaders in veterinary clinics make sense of their experiences around responding to employee voice in the workplace?

## Materials and methods

### Study design

As the research questions seek to understand nuanced sense‐making from lived experience at both the individual and group levels, this study employed an exploratory phenomenological approach which is well suited to identifying shared meanings across the groups in the veterinary setting. Phenomenology represents a well‐established qualitative framework providing a strong methodological foundation for the study design and analysis.^22^ Ethics approval was granted by The University of Queensland's Human Ethics Committee in February 2022 (project number 2021/HE002632). Semistructured interviews ranging from 28 to 105 min were used to collect data between February and December 2022, with an honorarium of $40 provided to participants who completed interviews. Interviews were transcribed, deidentified and assigned an anonymised numerical identifier to reflect interviewee role and experience (EV for experienced veterinarian employee, RV for recent graduate veterinarian employee, VN for veterinary nurse or technician employee and L for clinic leader or owner). Results from this study have been reported in line with the COREQ guidelines^23^ for qualitative analysis to ensure transparency and rigor of process.

### Methodological approach

A phenomenological approach, Interpretative Phenomenological Analysis (IPA),^24^, ^25^ was used to explore how participants experienced, interpreted and made meaning of speaking up (employees) or responding to voice (leaders) in Australian veterinary clinics. IPA has been used across many disciplines, including medicine,^26^ veterinary science^27^, ^28^ and psychology^29^ to understand lived experiences of various phenomena. This methodology was chosen as it strongly foregrounds participants' voices allowing a deep understanding of their perspective, while acknowledging the subjectivity of the researchers' interpretation. Hence, a crucial element of IPA is the process of *bracketing*, where the researcher is aware of, and puts aside as far as possible, their own experiences, knowledge and lens (*forestructures*) throughout the data analysis to ensure an analysis faithful to participants' voices.^25^ IPA methodology therefore provides inherent analytic rigor, an important consideration given the researchers' proximity in the veterinary setting. In view of the interpretative work involved in qualitative research, it is appropriate to describe the researchers involved in the study.^23^, ^30^ Our research team consists of five members. Researcher 1 (HG) is a veterinarian with a background of 20 years' experience in general practice veterinary clinics and was a PhD candidate at the time of the study researching how to improve retention of staff in veterinary clinics. Researcher 2 (EK) is a university academic skills advisor, qualitative researcher and has a background in clinical veterinary work and clinic ownership. Researcher 3 (AN) is a university academic in the field of organisational psychology with no prior involvement with veterinary research. Researcher 4 (DP) is a clinical veterinarian holding a leadership position at a university veterinary clinic. Researcher 5 (RS) is a clinical academic, psychosocial science researcher and experienced veterinary nurse. All researchers are cisgender female.

### Participants

In line with IPA methodology, a purposeful sampling strategy for maximal variability was employed for this study to achieve a diverse range of perspectives across roles, locations and clinics within the Australian veterinary profession and ensure depth and complexity in the data.^22^, ^24^ Anyone over the age of 18 who was working in or owning an Australian general practice veterinary clinic at the time of the study was deemed eligible to take part. Participants were recruited via self‐selection in response to advertisements on social media veterinary groups, through professional networks, via discussions at veterinary events, and snowball sampling with participants recommending other potential participants. All participants were provided with participant information sheets prior to interviews. In line with IPA methodology, subgroup sizes (3 to 9 participants) supported rich, in‐depth data and thematic convergence and divergence.^25^, ^31^ Recruitment was concluded at 24 interviews as the dataset as a whole was judged to provide adequate material to maintain the idiographic focus on individual experience while also allowing meaningful cross‐case interpretation.^25^, ^32^

### Interviews

Twenty‐four semistructured interviews were undertaken using interview guides developed specifically for the study in alignment with qualitative interviewing principles.^24^, ^33^ Five pilot interviews were undertaken to refine the interview guides and for the researcher conducting the interviews (HG) to gain experience with IPA interview techniques including rapport, reflection, allowing time and flexibility and positioning the participant as the expert of their experiences.^25^ The interview guides included open‐ended questions about participants' memorable experiences around speaking up (employees) or responding to voice (leaders), both positive and negative, which allowed participants to meander while still gathering relevant data.^34^

Twenty‐two interviews were undertaken via Zoom videoconferencing and two were completed face‐to‐face in cafes. Handwritten notes were taken during the interviews to capture context and document nonverbal observations^35^ (for example, gestures, facial expressions), threads to follow, patterns or reflexivity notes such as challenges or emotions experienced during the interview process. All interviews were recorded then transcribed clean verbatim and any identifiers removed. Member‐checking was undertaken with an interview summary developed and provided to participants along with the deidentified transcription to confirm accuracy and the researcher's understanding of the themes and interpretations of their experiences.^36^ Two participants suggested minor corrections which were incorporated prior to data analysis.

### Data analysis

Data analysis followed IPA processes whereby codes and themes were developed inductively from the data rather than deductively from theory to ensure participants' voices shaped the analysis.^37^ The first stage of analysis involved immersion in the data. This began during the transcription process of listening and relistening to the interviews, followed by several readings of the transcriptions. Each deidentified transcription was uploaded into Microsoft OneNote for Windows 10 (Version 16001.14326.22348.0) into a three‐column template to facilitate IPA coding processes. Each transcription was coded with a line‐by‐line analysis allowing initial exploratory noting of ideas, interesting phrases and repeated words. Interpretative work then began by developing experiential statements that drew out meaning making.^24^, ^25^ The experiential statements were then clustered into groups, noting convergence and divergence, becoming Personal Experiential Themes (PETs).^24^, ^25^ A PET table was developed for each participant. The PET tables were then used in cross‐case analyses for each group (leaders, employees), and additionally for each subgroup of employees (experienced vets over 10 years of experience, recent graduate vets up to 5 years of experience and veterinary nurses or technicians). Theme clustering and visual mapping were used to identify relationships between themes and develop Group Experiential Theme tables for each group.^24^, ^25^ Due to word limitations only the leader and employee group analyses are presented here.

Data analysis took place over an 11‐month period during 2023, with revisiting and refining as ongoing reflection occurred during the write‐up and feedback process over the following 18 months, as expected in the hermeneutic circle central to phenomenology.^25^, ^38^ As it is desirable to have diverse perspectives in qualitative analysis,^25^, ^39^ research team members RS, EK and AN also analysed two interviews each, with a total of six interviews for comparison. The research team met monthly throughout the analysis where interpretations and themes were discussed and either integrated into the analysis or collaboratively excluded, with an ongoing focus on participants' voices and reflexivity rather than purely intercoder reliability.

## Results

Table 1 Summarises the demographic data for the study.

**Table 1:** Interviewee demographics.

	Clinic leader or owner	Employee
Total N = 24	9	15
Role/experience		
Veterinarian	9	12
<5 years (recent graduates)	0	6
>10 years (experienced)	9	6
Veterinary nurse or technician	0	3
Gender		
Male	6	3
Female	3	12
Location of clinic		
City	4	7
Regional	5	8
State		
Queensland	5	7
New South Wales	2	5
Victoria	2	1
Western Australia	0	1
Northern Territory	0	1
Clinic ownership model		
Independent	8	10
Corporate	1	5
Known to researcher	5	4

Group Experiential Themes from the leader cross‐case analyses are summarised in Table 2 and expanded below to illustrate how leaders made sense of their experiences in responding to employee voice.

**Table 2:** Group Experiential Theme table for leaders.

Starting from a default position You don't know what you don't know – entering the leadership role with limited training or experience Leader values setting the scene for leader approaches to voice Relentless pressure and responsibility affecting leader capacity to encourage and respond to voice
An evolution of leadership skills and approaches to voice Learning from trial and error Choosing to be open to change as a catalyst for upskilling Approaching voice and responding with heightened awareness and intention
Recognising the practical and symbolic value of employee voice and leader responsiveness interaction Employees' ideas can contribute to better ways of working Intentional leader responses can convey care and empowerment

### Leaders

#### Starting from a default position

Throughout the interviews, leaders described varying approaches to encouraging and responding to employee voice during their time in leadership roles, learning on the job as they went. L06's explanation of their initial approach to their leadership role was echoed by other leaders over the interviews.

I think you just muddle on through, like the majority of the profession, you do what you've seen done before and there's no real foundation why you do it, that's just because. L06

Muddling through meant that leaders defaulted to their familiar approach based on their values and held the same expectations for employees that they held for themselves. Some leaders valued bravery and assertiveness and this shaped their approach to employee voice.

I've always been very much well I'll sort myself out and if something hasn't suited me I try and address it and I expect that most vets should be smart enough and brave enough to do that. L02

Alternatively, several leaders valued empathy and naturally incorporated this into how they facilitated and responded to voice.

I guess the main thing is, particularly with the lower‐level staff, is having empathy for them about what it's actually taken for them to come to you about it. Like what level of frustration have they had about it, what level of concern have they had about it. And how do you make them feel better about it. L01

These divergent approaches to facilitating employee voice highlight how differences in underlying values shaped leaders' default attitudes and expectations in their leadership role.

Another universal challenge experienced by leaders was the relentless pressure in juggling their clinical and management tasks and having the ultimate responsibility for the business.

Regardless of different approaches to their leadership roles, a shared experience of leaders was finding time and energy to listen, reflect, respond and implement change, as noted by L04.

I don't have the time to do team meetings or any of that kind of s***. And yeh maybe, I'm sure people would appreciate it but I've got enough things to worry about. L04

Leaders pointed to how facilitating and responding to voice could be thwarted by their capacity to make it happen, with workload and responsibilities taking priority. Similarly, L03 described how tiredness cut into their ability to be their best self when responding to voice.

Am I present and ready to receive this or is this happening at the end of a very long day…. now the staff member's complaining….L03

L03 was aware that a lack of time and energy reduced their capacity to interpret voice positively, despite their best intentions. Leaders brought themselves to their role with divergence in how they approached voice opportunities. Innate values and previous experience formed the basis for their attitudes, however the next theme points to the evolution of values, self‐awareness and intentionality as they grew in their leadership roles.

#### An evolution of leadership skills and approaches to voice

After a period of time ‘muddling through’ their early leadership journey, most leaders spoke of realising at some point that they needed to upskill their leadership and sought training. Several leaders spoke of an employee incident or feedback that was a catalyst for their leadership evolution and re‐evaluating their approach to employee voice.

I guess you make a decision somewhere along the line and for me the feedback was you're an a******e, literally in those words said to a third party who was doing a staff feedback survey, ‘(L06) is an intimidating a******e’. And when you get that feedback you kind of have to look at it and you have to have some sense of realisation. L06

Having or developing positivity towards receiving suggestions and willingness to change meant leaders could graciously receive difficult feedback which allowed them to respond with openness and evolve how they lead their team. Another example of a catalyst for change came as L09 recounted being caught unawares by an outburst and resignation from a staff member.

She just got up and said I don't need this s*** and left. And I went, goodness, what has been going on that I am totally unaware of. I have no idea. And sort of within the week she was completely gone. L09

L09 shared insight from this experience: ‘I learned to nip things in the bud a lot quicker’. By deliberately responding to build confidence in voice and subsequent action, over time leaders developed an openness to others' ideas:

That's a part of understanding that you haven't got all the answers, and you've got to listen to other people and see where their stuff fits into your plan. L09

As leaders evolved they became more intentional in how they managed and responded to employee voice, and for some, increasingly recognising the value of encouraging employees to speak up.

#### Recognising the practical and symbolic value of the employee voice and leader responsiveness interaction

From their baseline, or along their leadership evolution, leaders recognised the value of employee contributions and made attempts to respond positively even in the face of their own pressure. Leaders commented on the practical significance of responding positively to employee voice, with constructive suggestions leading to improvements in ways of working.

Usually the person who's identified something wrong also knows a better way to do it. The reason they're pissed off with it is – it shouldn't be like this, it should be like that. And so we're like hey great – you've got that idea, bring it out, let's talk about it, let's get it out in the open. L03

However, many also recognised the symbolic significance of their responses to employee voice, responding intentionally to ensure employees felt heard, valued and empowered. Several leaders acknowledged the importance of perception and attempted to respond so that employees felt heard and cared about.

We try and make sure people feel like they are actually listened to even if the answer is pretty much no. L07

You think you're listening but to them you're not… and perception's everything, it doesn't matter whether you are or not. Same with clients, doesn't matter whether you care or not, it matters if the client thinks you care. L08.

Similarly, L05 recognised the positive effects of a ‘yes’ in response to employee ideas and tried to instil a sense of empowerment to retain staff.

I don't say no very often, I don't come and trump them very often… If they keep feeling that I keep going Nup, nup, then you're not going to keep them for very long are you. L05

Overall, these experiences tell the story of leaders doing their best to respond to employee voice in challenging circumstances including relentless pressure, heavy responsibility, and limited capacity. Leaders' differing values resulted in varied approaches to employee voice, although as leaders evolved many strategically adapted their approaches to improve outcomes for their staff and business.

### Employees

Table 3 summarises the Group Experiential Themes for employees, with further explanation and quotes in later text demonstrating how veterinary employees made sense of their experiences around using upward voice in clinics and the response they received.

**Table 3:** Group Experiential Theme table for employees.

Internal thresholds and external barriers: Deciding to speak or stay silent I'm (not) one to speak up My boss is approachable/intimidating Voice is easy/a hassle in this workplace What was the point of that? The futility of unheard voice
Leader responses have a profound impact on employees' feelings about the clinic and the boss Feeling heard and valued versus feeling disillusioned and unsupported Unrewarded efforts: Why would I work for someone who treats me like that?

#### Internal thresholds and external barriers: deciding to speak or stay silent

Participants saw themselves as either one to speak up or not. They described personal characteristics such as shyness, preference for harmony, willingness to advocate for others, being proactive and cultural background as contributing to their natural approach to speaking up.

I didn't want to raise it because technically it's not my business and I just don't want to stir things up. E10RV

Combined with skills learned through life experiences, this formed the basis of their comfort with speaking up to their clinic leaders. However, despite this default position participants described different behaviours in different workplaces, with situational factors tipping the balance towards voice or silence.

Perceptions of clinic leaders played a major role in how comfortable employees were with speaking up in their workplaces. Observations and experiences of leaders' words and actions over time developed a pattern of either safety or wariness. Recent graduates and veterinary nurses or technicians were especially aware of the risks of voice in the face of a power differential, feeling unsure if their input was safe and welcome.

Like I don't want to be a new grad saying the man who's been a vet longer than I've been alive is wrong. E14RV

Recent graduate E08RV spoke of the many internal thresholds that needed to be crossed to raise voice in their previous workplace, with perceptions of favouritism and a fear of repercussions resulting in silence. However, in their next clinic, they raised suggestions with an approachable clinic owner perceived as open and willing to implement ideas.

(I suggested) to introduce a new drug into the clinic that I've used in the past … showed him the research, showed him specialist opinions and next week it was on the shelf kind of a thing. So very open to suggestions as long as you give him data. E08RV

In both clinics, E08RV had their defaults and learned skills. However, the environments and anticipated responses differed, leading to different choices around voice.

Employees were more inclined to voice when leaders were perceived to listen, care and take action. For example, a perceived caring manager prompted new graduate E10RV to overcome their default of quiet tolerance. They described choosing to speak up to the practice manager in their corporate clinic about an unsustainable workload rather than quitting.

I know that she cares about her staff, like she always wants the best for us so I know that she's willing to take feedback and she's happy to make changes… she really cares about our wellbeing. That's why I feel more comfortable raising these problems with her, because I know she's going to be fine with it. E10RV

Experienced vets also found voice ‘nerve‐wracking’ but the impact of approachability of leaders was pivotal; E07EV stated,

My current boss is quite active in seeking out feedback and making sure he presents himself as being there to listen…answer questions…give feedback and to be there for any concerns that we've got going on, so it doesn't feel like you're putting him out, he makes it feel like it's okay you can do it any time. It's easier…not quite as nerve wracking. E07EV

Employees' perceptions of their leaders as approachable or caring created a sense of safety in speaking up, contributing to decisions for voice instead of silence. Hence, while personal characteristics set the initial level of comfort for speaking up, the contextual factors, particularly having an approachable go‐to person in the position of power swung the balance towards voice over silence.

Participants described voice as ‘a battle’ and ‘a hassle’, requiring bravery and energy to speak up. Having clear and accessible voice channels in place provided a pathway that made the practicalities of voice easier but also showed that employee input was welcome. E07EV described how the presence of voice channels made it easier to raise the ‘little things’. They went on to reflect on the outcome of a lack of voice opportunities in a previous clinic.

The bosses at my last practice… they didn't invite feedback in the first place so the first they knew that someone wasn't happy and planning to leave was the resignation letter… it didn't have to happen, I knew the staff and they just wanted a conversation, wanted to feel valued. E07EV

Similarly, E08RV spoke of a lack of voice opportunities in their previous workplace contributing to low morale and dissatisfaction, eventually seeking out a workplace that valued communication.

Rumours started floating around and that just brought general morale down. And I think when there's such opaque management and lack of ability to give feedback I think resentment just starts building really quickly. E08RV

A lack of voice channels coupled with short‐term contracts also contributed to ambivalence about improvements in clinics for locum vet E05EV. They rarely saw the clinic owner, sending messages through the nursing staff if required, and tended to ‘put up’ with issues rather than raise them due to the short‐term nature of the work.

You're not really working with the boss. But then it can be hard to get that feedback through. Although when you're doing locums it's like oh well, I don't have to put up with this for too long. E05EV

In this situation, a ‘don't ask, don't tell’ approach existed, with the lack of voice channels and ambivalence leading to missed opportunities for improvements.

These experiences warn of the risks of leaving voice to chance and suggest that employees view the presence of formal voice channels as indications that their voice is welcome and worthwhile, creating a pathway for informal voice when required. However, voice channels were only effective when a response was received.

After the effort required to speak up, participants described a sense of futility when messages were lost or ineffective.

(It takes) a lot of courage and mental energy to give feedback and then sometimes it'll either get dismissed or you won't ever hear about it again and it kind of feels like well what was the point of that? E13VN

Experienced clinician E01EV described the difficulty in progressing urgent messages up through management layers in a previous corporate clinic and lacking any authority to solve the issue themselves.

You could talk, you could raise concerns but that was as far as it went if you were able to get onto somebody… Anything that costs money has to go up the chain and they have to talk about it and then back down the chain and I guess they're not necessarily there on the floor to see the impact that it's having. E01EV

Communication proved difficult with this disconnection, and a lack of contact regarding urgent concerns created stress, frustration and eventual exit from the clinic for E01EV.

However, voice could feel futile even in smaller clinics. E15VN described unsuccessfully and repeatedly trying to get a message through to the clinic owner in a privately owned multibranch clinic about an ongoing problem.

The other day I saw he has like fifty‐nine unread emails and I'm 100% sure that some of them are mine… yeah he says we can email with feedback, but a lot of the times it just gets lost in the pile. E15VN

When voice was ineffective a sense of frustration, powerlessness and futility developed, contributing to a feedback loop for future silence over voice.

#### Leader responses had profound impacts on how employees felt about their leader and their workplace

Leader responses to voice attempts were pivotal in how employees perceived the safety and effectiveness of voice at the time, but over time also built up a picture for employees of whether they felt cared about, valued and wanted to stay in that clinic. When employees perceived positive leader responses, they felt heard, valued and respected, with these participants speaking positively about their clinic and their boss. Conversely, employees who perceived negative responses to their voice attempts described frustration and anger, feeling disrespected and disillusioned.

Previous experiences provided a yardstick for how the response was perceived, with E01EV appreciating their current leader's ‘fair’ approach in the context of being ‘shut down’ by previous bosses.

It's comfort in knowing that you're going to get an open and fair discussion on whatever it is that you bring up where you're not just going to get shut down. Because I have been in the past where the response has been, from bosses, ‘do you want a job or not?’ E01EV

E01EV spoke positively about their current clinic leader, felt that their boss ‘had their back’ and spoke with loyalty about their workplace. Similarly, E10RV described their gratitude for the response and action from their practice manager after raising concerns.

I had a chat, a long chat with her about all these things and then she ended up…like I'm really grateful that she actually listened and then it did improve. E10RV

The positivity and appreciation expressed by employees whose bosses were perceived to listen to concerns, respond and take action, contrasted with the frustration, resentment and anger expressed by employees as they recounted experiences of perceived dismissive, ineffective or ‘victim‐blaming’ responses from clinic leaders. When describing experiences around negative leader responses, employee language became strong and emotional, for example ‘incredibly frustrated’, ‘unsupported’, ‘disrespected’, ‘unappreciated’, ‘isolating’, ‘unfair’ and ‘disheartening’, reflecting their sense of powerlessness and resentment.

Being ‘blamed’ by clinic leaders for issues raised resulted in unresolved concerns and frustration. When E09RV raised concerns with their clinic owners, the response failed to address the issue and was actually counterproductive.

Their response was more of a we're a family, we all have to work together, you shouldn't be complaining about having to pick up slack essentially, so it was just sort of dismissed as me coming across as being lazy I guess as opposed to the real issue which was having things be done preferably the first time… kind of got thrown back in my face, I was like I don't want to bring up anything else if there are any issues, just learnt to tough it out… I guess I just got very bitter. E09RV

For these employees, their experience of leader responses felt dismissive and blaming rather than addressing the underlying issue, resulting in silence, resentful tolerance and bitterness. This strong emotional response was particularly evident with employees who were showing initiative and moving beyond the confines of their role. E15VN described their frustration with the clinic owner's lack of support when concerns were raised, and the disillusionment and doubt that ensued when they felt their additional efforts for the clinic were undervalued.

I really feel like I'm not supported here and I'm not appreciated, and I do all this work. And I'm really easy, if there's an extra shift that needs to be covered, like I'll work extra hours, if you know the on‐call nurse for some reason doesn't pick up her phone, like I'll come in. And then like to get a kick in the gut like that … I was like I'm not sure if I want to work with someone who treats me that way. E15VN

Employees placed meaning on leader responses, determining if they were valued, supported and appreciated in their workplace. This was especially true for employees going above and beyond who wanted to feel valued and appreciated by their clinic leaders.

## Discussion

This study yields several important insights.

Firstly, the findings illuminate the critical impact the employee voice and leader responsiveness interaction can have in veterinary workplaces. At the organisation level, the interaction bridges disconnection between the frontline and upper levels of management, allowing correction of issues and improvements to processes. At the individual level, the interaction has a profound impact on employees' attitudes towards their leaders and clinics, ultimately contributing to decisions to stay or leave. The loyalty, empowerment and positivity described by employees who perceived positive responses from their clinic leaders suggests that these employees are the ‘enthusiastic stayers’.^5^

The findings also revealed that no news may not be good news. Employee voice is an optional behaviour^16^ and employees overcome internal thresholds and perceived external barriers to speak up. In this study, if employees did speak up there were challenges for leaders in responding constructively to employee voice due to time, energy, skills and values. Additionally, a feedback loop developed over time, with perceived positive (constructive) leader responses promoting future voice, and perceived negative (destructive) leader responses increasing the perception of futility and risk, promoting silence. Unfortunately the feedback loop from negative (destructive) leader responses encouraged future silence, thwarting the communication that could resolve a mismatch in expectations or prevent disillusionment. Hence, a destructive (blaming, dismissive) leader response to employee voice was not just a missed opportunity; it could actively disengage and silence enthusiastic employees. Silence or destructive leader responses also had implications for team dynamics, with resentment and low morale developing within the team in the face of unresolved concerns and a sense of powerlessness. The disparity between leader and employee perspectives on the ease and safety of voice suggests that the disconnect may be less visible from a position of power. This aligns with recent research into veterinary attitudes to safety that identified a potential disconnect between managerial perspectives and the lived experiences of clinical staff, suggesting improved communication between the two could improve safety climate.^40^ In light of the demonstrated importance of the employee voice and leader responsiveness interaction for outcomes in veterinary clinics, it is worthwhile considering how best to facilitate early, proactive voice met with timely and constructive leader responses.

In this study both leaders and employees described an evolution or upskilling process with learned skills assisting them to speak up (employees) and respond to voice (leaders). Awareness and skills helped people move past their default baseline into a more intentional position where they could use their voice more effectively or respond more thoughtfully and with an openness to ideas. However, in any workplace there is shared responsibility between the individual and the organisation; hence, it is crucial to consider organisational factors in addition to personal skills training. At the organisational level, this prompts consideration of how to implement processes that decrease the perceived costs of speaking up (risk, futility, effort) and increase the perceived benefits of voice (action, resolved concerns). Deeper understanding of these factors is needed, particularly in light of existing literature that demonstrates that employees instinctively view voice as risky, and it is challenging to enable them to speak up to those in power, suggesting that it takes continuous work to invite and reward speaking up.^41^ Results of this study reveal that leaders' responses and behaviours in their position of power played a critical role in how safe employees felt in speaking up. This buttresses well‐established research demonstrating that a sense of being able to speak up without fear of repercussions, one facet of psychological safety,^42^ is necessary for employee voice attempts. Therefore, this invites reflection by those in veterinary leadership roles on how their leadership style and behaviours may impact the work environment and staff perceptions. This is particularly important in light of the ‘learn on the job’ approach universally described by leaders in this study and in previous veterinary research^27^ whereby leaders experience a learning curve which may challenge them, and their staff. The relentless pressure and workload collectively described by leaders in this study is also concerning; a meta‐analytic review identified that leader stress was associated with negative (abusive) leadership behaviours as cognitive and emotional resources became drained.^43^ In the face of chronic stress and variable support, veterinary leaders face a considerable challenge to ensure good intentions translate into ongoing action.

Processes for voice differed between clinics; however, this study found a surprising commonality. In both independently owned clinics and corporate clinics, employees experienced frustration and disillusionment at lost messages and destructive responses from leaders, while in some clinics employees felt heard and supported. In this study, having an approachable, supportive person in a position to resolve concerns mattered more than the type of clinic. This contrasts with recent research that suggests that those working in independently owned clinics in the United States felt more emotionally supported than those working in corporate clinics.^44^ The findings from this study suggest there is an opportunity for organisations, both small and large, to improve outcomes by having an approachable go‐to person with the skills to respond constructively to voice and the power to implement change.

## Limitations

Despite its contributions, this study acknowledges some limitations. Due to including self‐selection as a sampling strategy, it is possible that those with strong views on the topic chose to participate, including those stemming from negative experiences. However, both positive and negative experiences were discussed during interviews to garner balanced perspectives across the dataset.

Additionally, several recent graduate veterinarians worked in different clinics under the same corporate umbrella and heard about the research through a supportive mentor within the organisation but external to their clinics. Hence, it is likely that their interviews represent perspectives of one, not all, corporate experience. However, this was also considered a strength in the study, providing a counterview of other participants where current and previous corporate experiences were expressed, suggesting there are ways to mitigate negative perceptions of working in corporate clinics.

Furthermore, only one leader from a corporate clinic was recruited. Clinic ownership model was included as a demographic iteratively during the analysis as multiple employees offered this information as a relevant consideration. Leaders did not raise the ownership model as relevant to their experience; however, this may reflect the recruitment process by role, and it is possible that there are differences in leader experiences responding to employee voice between independently owned and corporate clinics that were not captured in this study.

Finally, while not a limitation, it should be emphasised that this study, as with all qualitative research, did not use a random representative sample with the intention to generalise to the broader population. Rather, it targeted a group with experience in the phenomenon of interest, providing depth in the data based on human perceptions of their lived experiences. As such, the findings may be considered transferable to similar populations with similar contexts, for example, veterinary populations across Western societies, or other caring professions in similar private practice business models, but care should be taken generalising more broadly.

## Conclusion

This study found that when employees choose early proactive voice over silence, and leaders respond constructively, there are both practical outcomes (concerns are acknowledged/addressed) and relational outcomes (employees feeling empowered and valued) with the interaction associated with positive outcomes of employee loyalty and enthusiasm, supporting ‘enthusiastic staying’ in veterinary clinics. It points to an opportunity for clinics to improve outcomes by enhancing capabilities for both employees and leaders around speaking up and responding. Organisations and businesses can consider how they can ensure voice is perceived as safe, easy and effective and, crucially, ensure leaders are equipped with the support, skills and motivation to respond appropriately. Further research is needed to provide clarity around the organisational‐level conditions in veterinary clinics that best facilitate employee voice and allow leaders to respond in a timely and constructive manner.

## Conflict of interest and sources of funding

The authors have no conflicts of interest to declare.

## Data Availability

The data that support the findings of this study are available on request from the corresponding author. The data are not publicly available due to privacy or ethical restrictions.
